# The Transcription Factor YY1 Is a Novel Substrate for Aurora B Kinase at G2/M Transition of the Cell Cycle

**DOI:** 10.1371/journal.pone.0050645

**Published:** 2012-11-30

**Authors:** Ari Kassardjian, Raed Rizkallah, Sarah Riman, Samuel H. Renfro, Karen E. Alexander, Myra M. Hurt

**Affiliations:** 1 Department of Biomedical Sciences, Florida State University, Tallahassee, Florida, United States of America; 2 Institute of Molecular Biophysics, Florida State University, Tallahassee, Florida, United States of America; 3 Department of Biochemistry, Molecular Biology and Biophysics, University of Minnesota, Minneapolis, United States of America; North Carolina State University, United States of America

## Abstract

Yin Yang 1 (YY1) is a ubiquitously expressed and highly conserved multifunctional transcription factor that is involved in a variety of cellular processes. Many YY1-regulated genes have crucial roles in cell proliferation, differentiation, apoptosis, and cell cycle regulation. Numerous mechanisms have been shown to regulate the function of YY1, such as DNA binding affinity, subcellular localization, and posttranslational modification including phosphorylation. Polo-like kinase 1(Plk1) and Casein kinase 2α (CK2 α) were the first two kinases identified to phosphorylate YY1. In this study, we identify a third kinase. We report that YY1 is a novel substrate of the Aurora B kinase both in vitro and in vivo. Serine 184 phosphorylation of YY1 by Aurora B is cell cycle regulated and peaks at G2/M and is rapidly dephosphorylated, likely by protein phosphatase 1 (PP1) as the cells enter G1. Aurora A and Aurora C can also phosphorylate YY1 in vitro, but at serine/threonine residues other than serine 184. We present evidence that phosphorylation of YY1 in the central glycine/alanine (G/A)-rich region is important for DNA binding activity, with a potential phosphorylation/acetylation interplay regulating YY1 function. Given their importance in mitosis and overexpression in human cancers, Aurora kinases have been identified as promising therapeutic targets. Increasing our understanding of Aurora substrates will add to the understanding of their signaling pathways.

## Introduction

The zinc finger-containing transcription factor YY1 is a ubiquitously expressed multifunctional protein that is highly conserved among animal species. It has been shown to be the vertebrate homolog of the *Drosophila melanogaster* polycomb group protein Pleiohomeotic (Pho) [1]. As a transcription factor, YY1 regulates the expression of many genes that are critical for embryogenesis, differentiation, replication, cellular proliferation and apoptosis (reviewed in [2], [3]). Total ablation of the YY1 gene in mice causes embryonic lethality at the peri-implantation stage, while disruption of one allele caused significant growth retardation and developmental abnormalities, reflecting the essential role of YY1 [4]. At the cellular level, knockdown of YY1 slows cell cycle progression and cell proliferation and causes an accumulation of multinucleated cells with defects in cytokinesis [5]. Depletion of YY1 has also been shown to reduce the expression of critical kinases that regulate mitosis and cytokinesis, such as Aurora A, Aurora B and Polo-like kinase 1 (Plk1) [5]. In addition, genome-wide analysis of depleted YY1 mouse embryonic fibroblasts (MEFs) identified over 500 putative YY1 target genes [5]. Even though a wealth of data exists on the regulation of YY1 target genes and the role of YY1 throughout the cell cycle, little is known on how the YY1 protein itself is controlled or the upstream signaling pathways that regulate its function.

The expression of YY1 protein levels has been reported to be constant across the cell cycle [6], [7]. However, under certain physiological conditions, YY1 protein levels can be up regulated by the addition of growth factors, such as insulin-like growth factor-1 (IGF-1), fibroblast growth factor-2 (FGF-2) [8], [9], and by the cytokine TNF-α [10]. YY1 expression is stimulated by the transcription factor NF-kappa B, which directly binds to the YY1 promoter [11]. During skeletal myogenesis, YY1 can be down regulated by miR-29, which targets the 3′-UTR of YY1 mRNA and blocks translation [12]. Raf kinase inhibitor protein (RKIP), a metastasis suppressor gene can also down regulate YY1 expression through inhibiting its transcription [13]. YY1 protein levels have been shown to be deregulated during tumorigenesis and elevated YY1 levels have been detected in many types of cancers [3], [14], [15].

YY1 is also regulated by post-translational modifications. Multiple residues on YY1 are targets of post-translational modification, including, S-nitrosation [16], acetylation [17], [18], O-linked glycosylation [19], sumoylation [20], and poly(ADP-ribosyl)ation [21], [22], all of which regulate the function and activity of YY1. More recently, we identified and mapped multiple phosphorylation sites in YY1, including, threonine 39, serine 118, serine 247, threonine 348 and threonine 378 [7], [23]–[25]. The first kinase proven to phosphorylate YY1 in vivo was Plk1, which phosphorylates threonine 39 during G2/M stage of the cell cycle [25]. CK2α is another kinase identified as constitutively phosphorylating YY1 at serine 118. This modification protects YY1 cleavage by caspase 7 during apoptosis [23]. Our lab also reported that phosphorylation of YY1 in the DNA binding domain (threonine 348 and threonine 378) during mitosis abolishes its DNA binding activity [7].

We provide evidence here that a third kinase, the Aurora B kinase of the Aurora kinase family, also phosphorylates YY1 in vitro and in vivo. The Aurora kinases constitute a family of conserved serine/threonine kinases that are involved in cell cycle regulation and play critical roles in mitosis [26]. They were first discovered in a screen to identify genes involved in mitotic spindle function in *Drosophila* [27]. The mammalian genome contains three members of the Aurora kinase family, Aurora A, B, and C. Aurora B mRNA and protein expression levels, as well as Aurora A levels, peak at G2/M stage of the cell cycle and maximal kinase activity is reached during metaphase [28], [29]. Aurora B plays a critical role in the regulation of spindle assembly checkpoint pathway, chromosome condensation and biorientation, microtubule dynamics and cytokinesis (reviewed in [26], [30]). Aberrant expression of the Aurora kinases has been shown to cause cellular transformation and genetic instability. Given their importance in mitosis and overexpression in human cancers [31], Aurora kinases have been identified as promising therapeutic targets, and extensive effort has been devoted to developing inhibitors of these kinases and understanding their signaling pathways [32], [33].

An array of Aurora B substrates has been identified so far, including, histone proteins, spindle check point proteins, cytoskeletal proteins and enzymes [34]–[41]. More recently, the transcription factor p53 and the tumor suppressor Retinoblastoma protein (Rb) were shown to be targets of Aurora B. By directly phosphorylating Rb, Aurora B was shown to regulate the post-mitotic checkpoint [42]. Aurora B was also found to phosphorylate multiple sites in the DNA binding domain of p53, which significantly impaired p53 transcriptional activity [43]. These studies show a wide range of Aurora B substrates, including important cell cycle regulators and transcription factors.

Here, we report that the transcription factor YY1 is a novel substrate for the Aurora B kinase. Aurora B phosphorylates YY1 at serine 184 in the central regulatory domain. We show that this phosphorylation is cell cycle regulated, peaking at G2/M, which correlates with an increase in Aurora B protein expression and kinase activity. YY1 is rapidly dephosphorylated as cells exit mitosis and enter G1. Serine 184 is dephosphorylated by the Aurora B antagonist, protein phosphatase 1 (PP1), but not protein phosphatase 2A (PP2A), in vitro. In addition, phosphorylation in the central regulatory domain appears to play a critical role in YY1 DNA binding activity, implicating the Aurora B kinase in transcriptional control of YY1 regulated genes at the entry of mitosis.

## Results

### Characterizing the Anti-phospho-serine184 Antibody

YY1 is a ubiquitously expressed multifunctional transcription factor that belongs to the Polycomb Group protein family. It is involved in the transcriptional control of a large number of mammalian genes; therefore, understanding the phosphorylation signaling pathway that regulates YY1 function is crucial. Previously, we identified multiple phosphorylation sites on YY1 [7], . However, another novel phosphorylation site (Serine 184) was identified by a global mass spectrometry-based identification technique [44].

**Figure 1:**
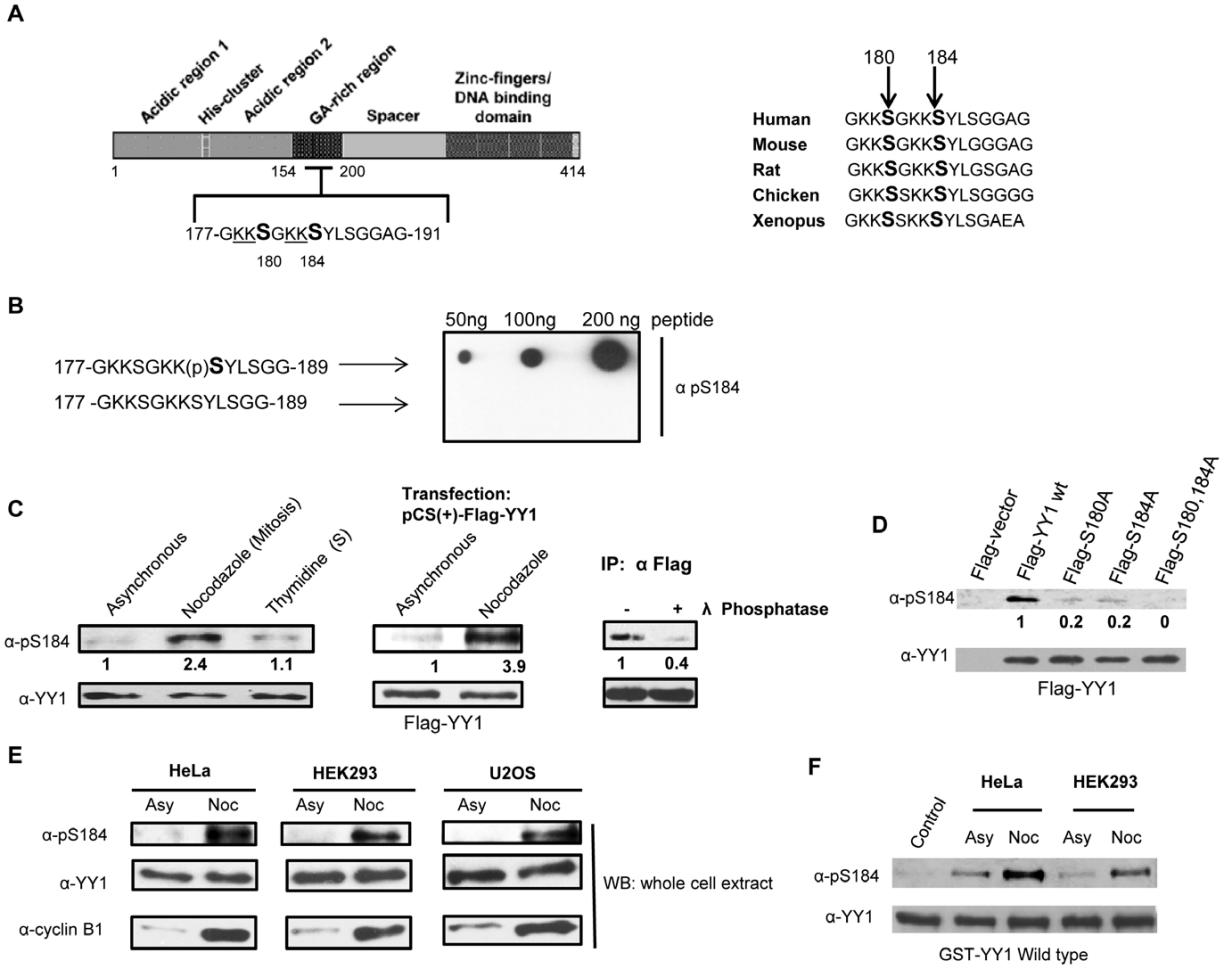
Phosphorylation of YY1 in nocodazole blocked extracts is detected by anti-phospho-S184 antibody. (**A**) Diagram showing the different domains of the YY1 protein. Amino acid residues 177–191 are shown, which include serine 180 and 184, as indicated (left panel). Amino acid sequence alignment of residues 177–191 of human YY1 from different animal species, as indicated (right panel). (**B**) Dot blot assay of non-phosphorylated and serine 184 phosphorylated synthetic peptides of YY1 amino acid sequence 177 to 189 probed with anti-pS184. (**C**) Asynchronous, nocodazole treated (100 ng/ml for 18 hours) and thymidine treated (2.5 mM for 18 hours) HEK293 cell lysates were prepared, followed by Western blot. The blot was probed with anti-pS184 antibody with relative levels indicated below, then stripped and reprobed with anti-YY1 antibody (left panel). Western blot analysis of asynchronous and nocodazole treated HEK293 cells transiently transfected with Flag-YY1, probed with anti-pS184 antibody with relative levels indicated below, then stripped and reprobed with anti-YY1 antibody (middle panel). Flag-YY1 was immunoprecipitated from transiently transfected HEK293 cells treated with nocodazole. Flag-YY1 bound to anti-Flag mouse MAb cross-linked to resin beads was resuspended in phosphatase buffer, and incubated with (+) or without (-) λ-phosphatase at 30°C for 30 minutes, followed by a Western blot of the samples. The blot was probed with anti-pS184 antibody with relative levels indicated below, then stripped and reprobed with anti-YY1 (right panel) (**D**) Western blot analysis of nocodazole treated HEK293 cells transiently transfected with Flag- vector, Flag-YY1 wild type, Flag-YY1 S180A, Flag-YY1 S184A and Flag-YY1 S180,184A was probed with anti-pS184 antibody with relative levels indicated below, then stripped and reprobed with anti-YY1 antibody. (**E**) Asynchronous and nocodazole treated (Asy or Noc) HeLa, HEK293 and U2OS cell lysates were prepared, followed by Western blot analysis. The blot was probed with anti-pS184 antibody, then stripped and reprobed with anti-YY1 antibody followed by anti-cyclin B1 antibody to show proper mitotic synchrony (left panel). (**F**) Cold in vitro kinase assay reactions using Hela and HEK293 whole cell extracts (50 µg) as the kinase source. Both asynchronous (Asy) and nocodazole treated (Noc) extracts were used. Bacterially expressed GST-YY1 wild type bound to glutathione beads were used as substrate. The reactions were performed at 30°C for 45 minutes followed by Western blot analysis. The blot was probed with anti-pS184 antibody, then stripped and reprobed with anti-YY1 antibody to show equal GST-YY1 levels.

Serine 184 of YY1 is a residue located in the central regulatory domain of the protein (glycine/alanine rich region) (Fig. 1A). To better understand and characterize the phosphorylation of YY1 at serine 184, a rabbit polyclonal phospho-specific antibody (α-pS184) was developed against a synthetic peptide encompassing YY1 residues 177–189 and containing a phosphorylation on serine 184. To test the phospho-specificity of the anti-pS184 antibody, we performed a dot blot assay spotting synthetic non-phosphorylated and phosphorylated forms of the peptide onto a nitrocellulose membrane. The anti-pS184 antibody efficiently recognized only the phosphorylated form of the peptide (Fig. 1B).

To investigate the presence of this phosphorylation in vivo, we prepared cell lysates from HEK293 cells growing asynchronously, treated with nocodazole to arrest cells in mitosis, or treated with thymidine, blocking DNA replication and arresting cells at S stage of the cell cycle. The proteins in the extract were separated on an SDS-PAGE gel and transferred to a nitrocellulose membrane. The anti-pS184 antibody detected YY1 protein at the correct molecular weight, in extracts from nocodazole-treated mitotic HEK293 cells and significantly less so in both asynchronously growing and the thymidine-treated cells (Fig. 1C, left panel). The phospho-band was confirmed to be YY1 by reprobing the blot with anti-YY1 antibody (Fig. 1C, left panel). Transiently overexpressed Flag-YY1 in HEK293 cells further confirmed the presence of the phospho-signal as being that of phosphorylated YY1 protein, present at higher levels in mitotic cells compared to asynchronously growing cells (Fig. 1C, middle panel). To further validate the specificity of the anti-pS184 antibody and to verify that the phospho-antibody does not recognize non-phosphorylated YY1, Flag-YY1 was immunoprecipitated from transiently transfected HEK293 cells treated with nocodazole. Immunoprecipitated Flag-YY1 protein was then incubated with or without λ-phosphatase at 30°C for 30 minutes, followed by Western blot analysis with anti-pS184 antibody. We show that this signal is phospho-specific, since phosphatase treatment of the immunoprecipitated Flag-YY1 from nocodazole extracts abolished the signal (Fig. 1C, right panel).

### Anti-phospho-serine184 Antibody Specifically Recognizes Phosphorylation of YY1

We next mutated both serine 180 and 184 of YY1 individually or together into alanine, a non-phosphorylatable residue. We were also interested in studying phosphorylation at serine 180 because of the similarity of its surrounding amino acids (−3 to −1 positions) to that of serine 184 (Fig. 1A). To examine the specificity of the antibody to its target sequence in YY1, nocodazole treated HEK293 cells were transiently overexpressed with Flag- vector, Flag-YY1 wild type, Flag-YY1 S180A, Flag-YY1 S184A and Flag-YY1 S180,184A. Cell lysates were prepared and analyzed by Western blotting. Anti-pS184 antibody was able to detect phosphorylation at serine 184 in Flag-YY1 wild type protein as seen previously, however the single mutants of serine 180 and 184 showed significantly less phosphorylation compared to wild type. In addition, phosphorylation was completely abrogated in Flag-YY1 S180,184A double mutant protein (Fig. 1D), suggesting either that serine to alanine mutation of 180 affects the phosphorylation liability at serine 184 or both serine 180 and serine 184 are possibly phosphorylated in vivo.

YY1 is ubiquitously expressed in all tissues [45]. The presence of phosphorylation at serine 184 was observed in mitosis in three different cell lines derived from different human tissues: HEK293, HeLa and U2OS (Fig. 1E), suggesting a common regulatory mechanism of YY1. YY1 is also a highly conserved protein among animal species. Protein sequence alignment shows that serine 184 and the flanking amino acid region are evolutionarily conserved, particularly the positively charged lysine (K) residues at −1, −2, −5 and −6 positions relative to serine 184 (Fig. 1A). These lysine residues are likely a critical part of a sequence motif which may govern kinase-substrate recognition.

We next performed a cold in vitro kinase assay using both HeLa and HEK293 asynchronous and mitotic extracts supplemented with 3 mM ATP used as our kinase source. Bacterially expressed GST-YY1 wild type bound to glutathione beads were used as substrate. The results show that GST-YY1 wild type protein is phosphorylated at serine 184 to a greater extent when incubated with mitotic extracts compared to asynchronous extracts (Fig. 1F). These results confirm that serine 184 is phosphorylated both in vitro and in vivo by a kinase present or highly active in mitosis.

### Phosphorylation of YY1 at S184 Peaks at G2/M Stage of the Cell Cycle

**Figure 2:**
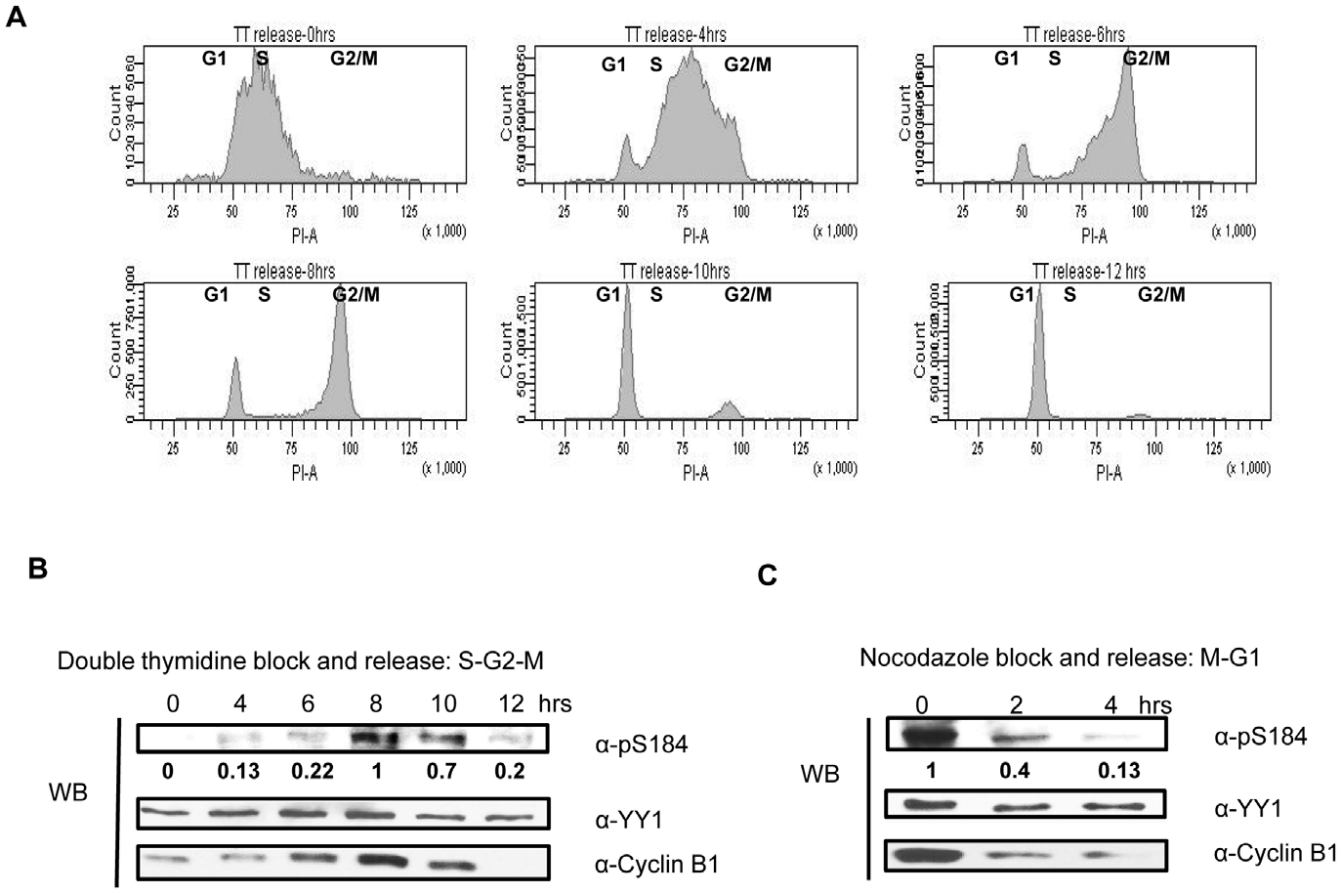
Serine 184 phosphorylation on YY1 peaks at the G2/M stage of the cell cycle. (**A**) Cell cycle progression of HEK293 cells released after double-thymidine (2.5 mM) block was analyzed by fluorescence-activated cell sorting. Cells were stained with propidium iodide to analyze DNA content. (**B**) HEK293 cells were synchronized at early S phase by double-thymidine block and then released into fresh media. Western blot was performed on HEK293 cell lysates collected at the indicated time points. The blot was probed with anti-pS184 antibody with relative levels indicated below, then anti-YY1 antibody, followed by anti-cyclin B1 antibody. (**C**) HEK293 cells were also synchronized in mitosis by nocodazole block (100 ng/ml) for 18 hours and then released into fresh media. Western blot was performed on HEK293 cell lysates collected at the indicated time points after nocodazole block and release. The blot was probed with anti-pS184 antibody with relative levels indicated below, then anti-YY1 antibody followed by anti-cyclin B1.

We have shown that phosphorylation on serine 184 is present in mitosis, but to have a better understanding of the timing of this modification in the cell cycle, we synchronized HEK293 cells using double thymidine block as described in the methods section. After the second thymidine block, cells were released into fresh media and samples were collected at the indicated time points for cell cycle analysis using propidium iodide staining followed by Fluorescence Activated Cell Sorting (FACS) analysis or for cell lysate preparation. Figure 2A shows the cell cycle distribution of cells as they start to progress from the thymidine block. Cells blocked with double-thymidine show early S-phase DNA content. Two and four hours after release, cells progressed into S-phase. At six hours, cells appear to have G2 level of DNA content, and at eight hours, they appear to be at late G2 and moving through G2/M into mitosis. At ten hours, most cells had exited mitosis and entered G1. Twelve hours after release, all cells were in G1 of the new cell cycle (Fig. 2A). Expression of cyclin B1 protein increases at the end of S-phase and accumulates at the G2/M boundary. In anaphase, cyclin B1 levels drop dramatically through rapid degradation, therefore cyclin B levels can be used as a cell cycle marker [46]. As observed in figure 2B, cyclin B1 levels increase and reach their highest point eight hours post release and then decrease dramatically, indicating that the cells proceeded from S phase to G2 and into mitosis. Western blot analysis using anti-pS184 antibody indicate that serine 184 is phosphorylated mainly at the eight hour time point in correlation with the peak of cyclin B1 levels and G2/M transition by FACS analysis (Fig. 2B).

HEK293 cells were also synchronized in mitosis by nocodazole block for 18 hours and then released into fresh media. Western blot analysis was performed on HEK293 cell lysates collected at the indicated time points after nocodazole block and release. At the zero hour time point when all the cells are arrested in mitosis, cyclin B1 levels are highest and decrease dramatically as the cells are released into fresh media (Fig. 2C), indicating that the cells proceeded from mitosis and into G1. Western blot analysis using anti-pS184 antibody indicate that serine 184 is rapidly dephosphorylated as the cells leave mitosis and enter into G1, indicating the presence of a highly active phosphatase and an inactivation and/or degradation of the kinase responsible for serine 184 phosphorylation.

### YY1 is a Substrate for the Aurora Kinases

Next, we were interested in identifying the kinase responsible for phosphorylation of YY1 at serine 184. Timing of the phosphorylation, which peaks at the G2/M stage of the cell cycle as well as the consensus phosphorylation site surrounding serine 184, directed us to the Aurora kinase family. The Aurora kinases are known to be highly active at G2/M [47]. Based on previously reported phosphorylation sites on Aurora substrates, a consensus phosphorylation motif has been established. In general, Aurora kinases phosphorylate target substrates that have basic amino acid residues from −1 to −3 positions relative to the phosphorylation site [48].

**Figure 3:**
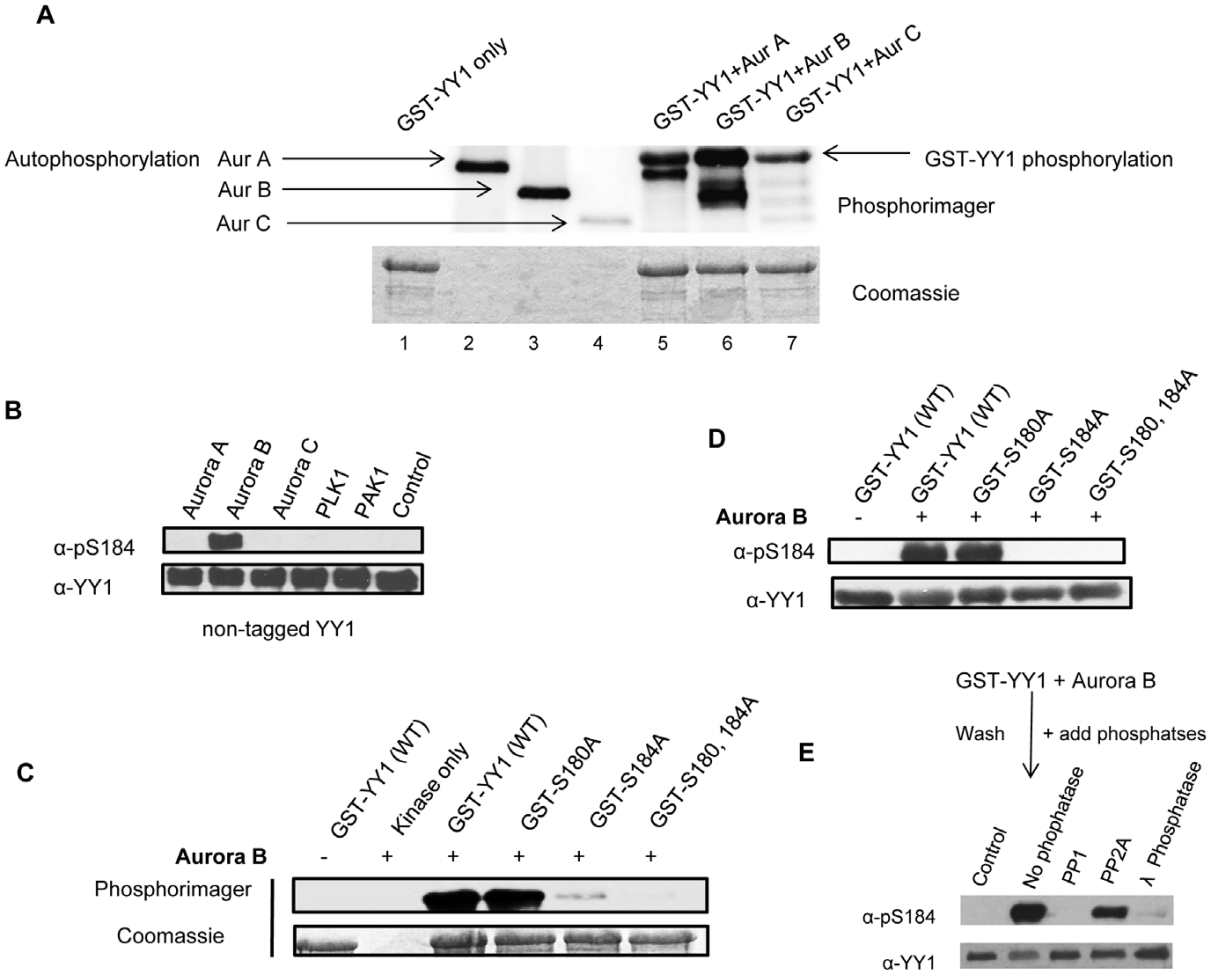
Aurora B phosphorylates YY1 at serine 184 in vitro. (**A**) Radioactive in vitro kinase assay using purified Aurora kinase isoforms and GST-YY1 as substrate. The kinase reactions include GST-YY1 only (no kinase), Aurora A, Aurora B and Aurora C only (no substrate) and GST-YY1 with each Aurora isoform. The reactions were performed at 30°C for 30 minutes. The reaction mixture was separated on a 10% SDS-PAGE gel and stained with Coomassie blue to visualize the protein bands and exposed to a phosphorimager screen. (**B**) Cold in vitro kinase assay reactions using purified Aurora A, Aurora B, Aurora C, Plk1 and PAK1 kinases and purified non-tagged YY1 as substrate. The reactions were performed at 30°C for 30 minutes followed by Western blot. The blot was probed with anti-pS184 antibody, then stripped and reprobed with anti-YY1 antibody. (**C**) Radioactive in vitro kinase assay using purified Aurora B kinase and GST-YY1 as substrate. The kinase reactions include GST-YY1 only (no kinase), Aurora B only (no substrate) and GSTY-YY1, GST-YY1 S180A, GST-YY1 S184A or GST-YY1 S180,184A with Aurora B. The reactions were performed as described for panel A. (**D**) Cold in vitro kinase assay reactions using purified Aurora B and GST-YY1 as substrate. The kinase reactions include GST-YY1 only (no kinase), Aurora B only (no substrate) and GST-YY1, GST-YY1 S180A, GST-YY1 S184A or GST-YY1 S180,184A with Aurora B. The reactions were performed at 30°C for 30 minutes followed by Western blot. The blot was probed with anti-pS184 antibody, then anti-YY1 antibody. (**E**) Cold in vitro kinase assay reactions using purified Aurora B and GST-YY1 as substrate. The kinase reactions were performed at 30°C for 30 minutes. After the reaction, GST-YY1 was washed with lysis buffer and resuspended in phosphatase buffer, and incubated with protein phosphatase 1 (PP1), protein phosphatase 2A (PP2A) or λ-phosphatase at 30°C for 30 minutes, followed by Western blot. The blot was probed with anti-pS184 antibody, then stripped and reprobed with anti-YY1.

To test if YY1 is a good substrate for the Aurora kinases, we performed a radioactive in vitro kinase assay using bacterially expressed GST-YY1 as substrate and purified Aurora A, Aurora B and Aurora C kinases (SignalChem). As shown in Figure 3A, all three isoforms of the Aurora kinases were able to efficiently phosphorylate GST-YY1 in vitro (lanes 5-7). GST-YY1 alone did not show any autophosphorylation (lane 1), however all three Aurora isoforms displayed autophosphorylation (lanes 2-4), as has been previously reported [49], [50].

### Aurora B Phosphorylates YY1 at Serine 184 in vitro

To identify the Aurora isoform that was specifically phosphorylating YY1 at serine 184, we performed a cold in vitro kinase assay using bacterially expressed non-tagged YY1 [25] as substrate and purified Aurora A, Aurora B and Aurora C. Also as control, we included the Polo like kinase1 (Plk1) and p21 activated kinase (PAK1) in the reactions, both of which are highly expressed and active during mitosis [51], [52]. The kinase reactions were then separated on a SDS-PAGE gel and transferred to a nitrocellulose membrane. The blot was probed with anti-pS184 antibody and only one band was detected, specifically in the lane where YY1 was incubated with Aurora B kinase. The blot was stripped and reprobed with anti-YY1 antibody to show equal YY1 levels. No other phospho-band was detected when YY1 was incubated with the other kinases (Fig. 3B), indicating that only Aurora B was able to phosphorylate YY1 at serine 184. Aurora A and Aurora C were phosphorylating other serine/threonine residue(s) on YY1. To further confirm these results, a radioactive in vitro kinase assay (Fig. 3C) and a cold in vitro kinase assay (Fig. 3D) were performed using the point mutants. Both kinase reactions included GST-YY1, GST-YY1 S180A, GST-YY1 S184A and GST-YY1 S180,184A incubated with Aurora B. The results show that serine 184 is the major site of phosphorylation by Aurora B. Aurora B also appears to phosphorylate serine 180 of YY1, but to a much lower extent in vitro (Fig. 3C). The main antagonist of Aurora B phosphorylation is PP1 [53]–[55]. After YY1 phosphorylation at serine 184 by Aurora B, the addition of purified PP1 can efficiently dephosphorylate serine 184, but not PP2A (Fig. 3E).

Based on the consensus phosphorylation site at serine 184 of YY1, two other mitotic kinases showed high probability for phosphorylating serine 184. We show that protein kinase A (PKA) and Rho-associated, coiled-coil containing protein kinase 1 (ROCK1) can also phosphorylate YY1 at serine 184 in vitro (Fig. S1A and S1B). However, treating HEK293 cells with chemical inhibitors for PKA or ROCK1 did not show a decrease in phosphorylation at serine 184 in vivo (data not shown). It is possible that PKA and ROCK1 may phosphorylate YY1 at serine 184 in other tissue types.

### Aurora B Interacts with and Phosphorylates YY1 in vivo

**Figure 4:**
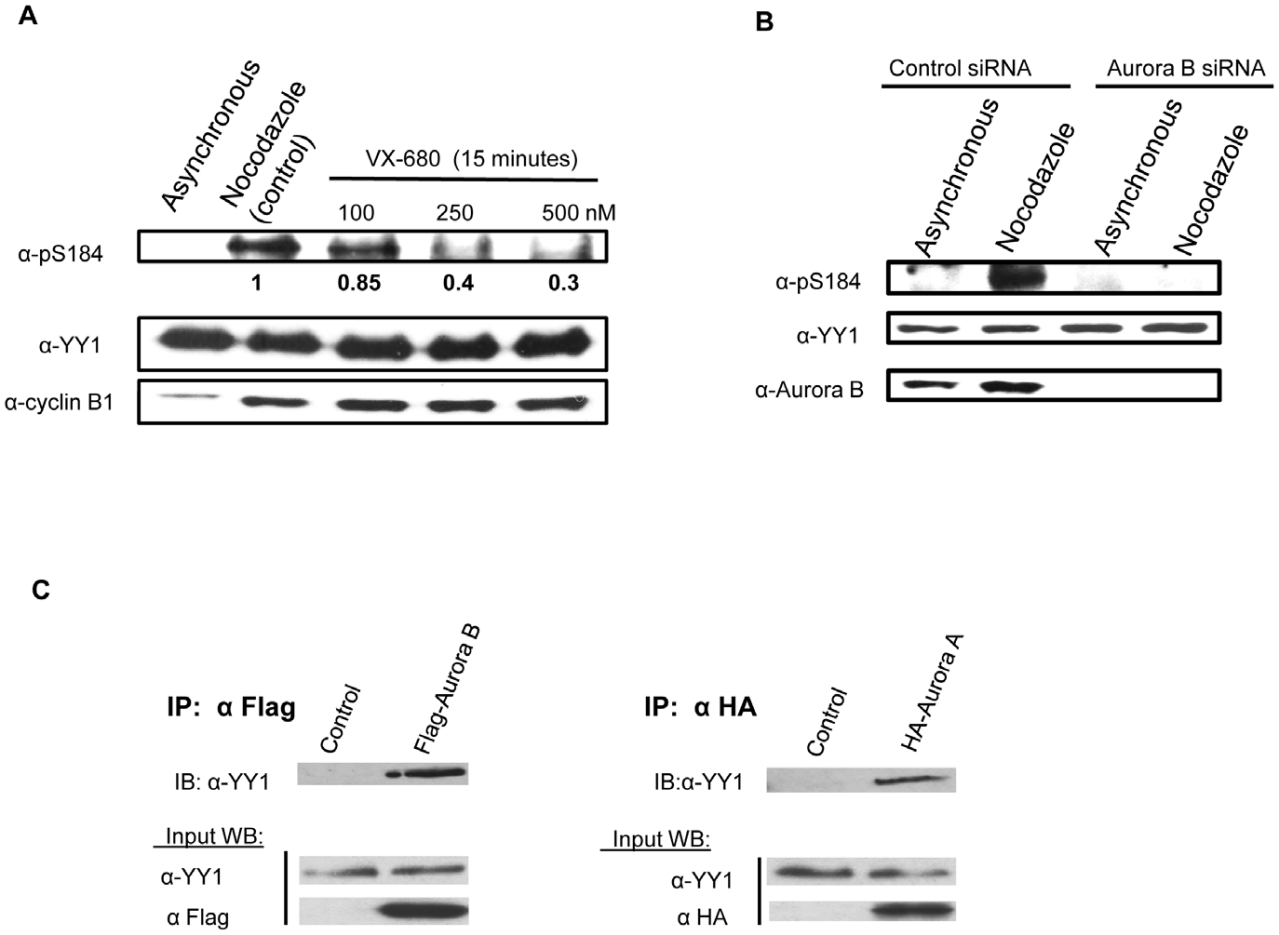
Aurora B phosphorylates YY1 at serine 184 in vivo. (**A**) HEK293 cells were synchronized in mitosis with nocodazole block for 17 hours. After the block, the cells were treated with the Aurora inhibitor VX-680, with the indicated concentrations for 15 minutes. Cell lysates were prepared, followed by Western blot. The blot was probed with anti-pS184 antibody with relative levels indicated below, then stripped and reprobed with anti-YY1 antibody followed by anti-cyclin B1antibody to show proper mitotic synchrony. (**B**) HEK293 cells were plated, cultured overnight, and then transfected with 20 nm control scrambled siRNA or Aurora B siRNA. After 48 hours of knockdown, the cells were lysed, and extracts were analyzed by Western blotting. The blot was probed with anti-pS184 antibody then stripped and reprobed with anti-YY1 antibody followed by anti-Aurora B antibody. (**C**) Co-immunoprecipitation of YY1 with Aurora A and Aurora B from HEK293 cells transiently transfected with HA-Aurora A, Flag-Aurora B and no transfection (control) followed by nocodazole block. Aurora A was immunoprecipitated using anti-HA antibody and Aurora B was immunoprecipitated using anti-Flag mouse MAb cross-linked to resin beads. Non-transfected cells were also immunprecipitated using anti-HA antibody and anti-Flag mouse MAb cross-linked to resin beads, which were used as a control for the specificity of the immunoprecipitation. Western blot analysis was performed on the immunoprecipitated samples and probed with anti-YY1 antibody. Input samples were probed with anti-YY1 antibody, anti-HA antibody and anti-Flag antibody.

To provide in vivo evidence that YY1 is a substrate for Aurora B, HEK293 cells were synchronized in mitosis with nocodazole block for 17 hours. After the block, cells were treated with VX-680, a potent and highly specific Aurora kinase inhibitor, at three different concentrations (100 nM, 250 nM and 500 nM) for 15 minutes. Cell lysates were prepared, followed by Western blot analysis. The blot was probed with anti-pS184 antibody, then stripped and reprobed with anti-YY1 antibody. The blot was also probed with anti-cyclin B1 antibody to show the cells were still in mitosis after drug treatment. Total YY1 protein levels were shown to be equal; however, the level of serine 184 phosphorylation significantly decreased upon addition of the Aurora kinase inhibitor (Fig. 4A). HEK293 cells were also transfected with 20 nM Aurora B siRNA (Fig. 4B). Nocodazole was added 30 hours after transfection for 18 hours for a total of 48 hour siRNA transfection. After 48 hours, the cells were lysed, and extracts were analyzed by Western blotting. The blot was probed with anti-pS184 antibody, then stripped and reprobed with anti-YY1 and anti-Aurora B antibody. Total YY1 protein levels were shown to be equal; however, Aurora B protein levels were completely knocked down in the Aurora B siRNA treated cells. In addition, serine 184 phosphorylation of YY1 was completely absent (Fig. 4B). As additional evidence that YY1 is a substrate for Aurora B in vivo, we performed a co-immunoprecipitation experiment using HEK293 cells transiently transfected with Flag-Aurora B and no transfection (control) followed by nocodazole block. We observed that endogenous YY1 was able to interact with Flag-Aurora B when pulling down the kinase using anti-Flag mouse MAb cross-linked to resin beads (Fig. 4C left panel). Similar results were also seen with Aurora A. Endogenous YY1 was able to interact with HA-Aurora A (Fig. 4C right panel). This is direct evidence for an in vivo physical interaction between YY1 and both Aurora A and Aurora B in mitosis, however only Aurora B was shown to phosphorylate YY1 at serine 184 (Fig. 3B). The amino acid residue(s) being phosphorylated by Aurora A and Aurora C on YY1 (Fig. 3A) will be further investigated in the future.

### Phosphorylation of YY1 in the Regulatory Domain Affects its DNA Binding Activity

**Figure 5:**
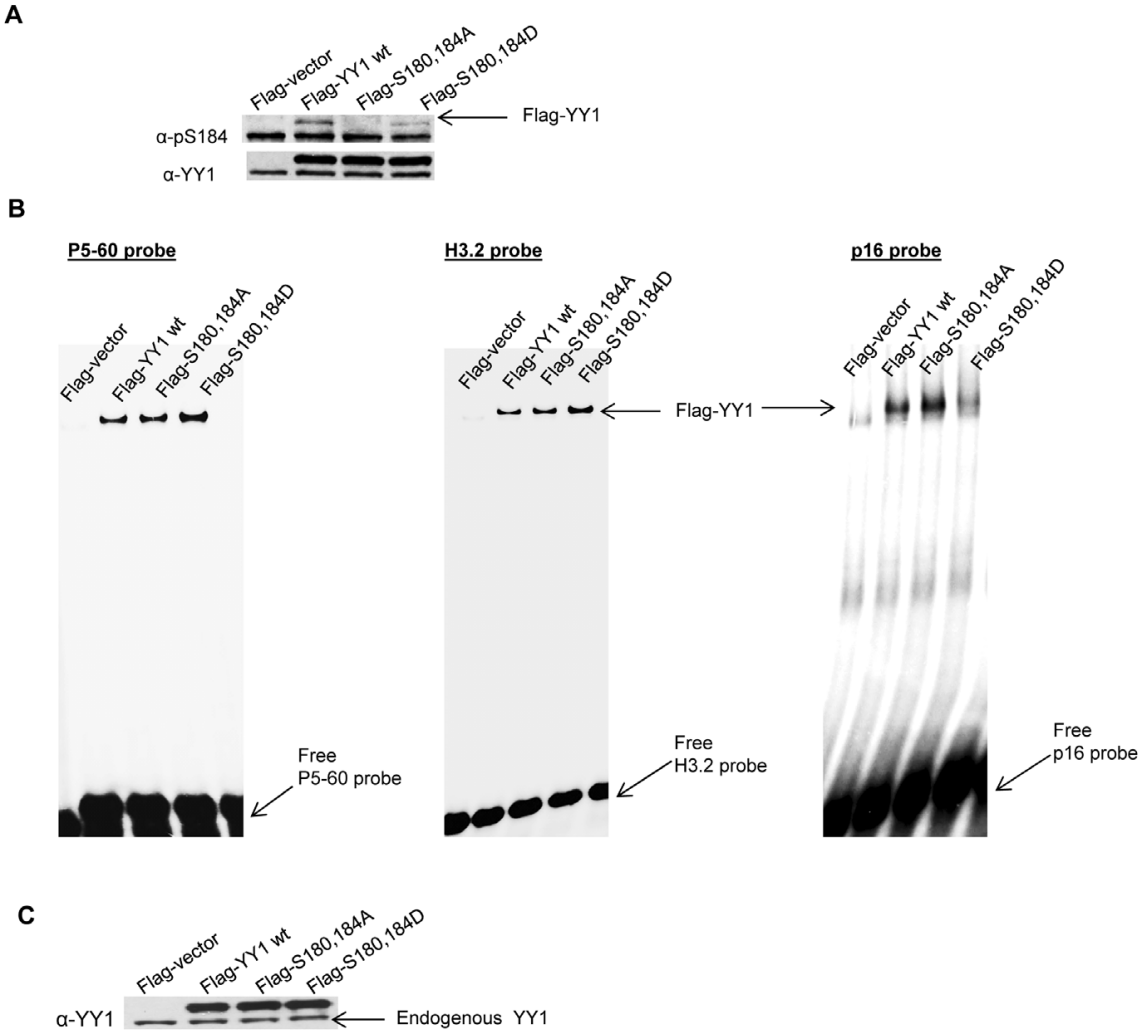
YY1 S180,184D phospho-mutant exhibits an increased DNA binding affinity in vitro. (**A**) Whole cell lysates were prepared from nococdazole treated HEK293 cells transiently transfected with Flag-vector, Flag-YY1 wild type, Flag-YY1 S180,184A and Flag-YY1 S180,184D followed by Western blot. The blot was probed with anti-pS184 antibody, then stripped and reprobed with anti-YY1 antibody. (**B**) Whole cell lysates were prepared from asynchronous HEK293 cells transiently transfected with Flag-vector, Flag-YY1 wild type, Flag-YY1 S180,184A and Flag-YY1 S180,184D. Cell lysates were used in an electrophoretic mobility shift assay (EMSA) using ^32^P-labeled H3.2 α, P5-60 and p16 DNA oligonucleotides as probes. (**C**) The same lysates used in the EMSA were also used in a Western blot probed with anti-YY1 antibody to show equal Flag-YY1 levels.

The addition of a phosphate group on a serine, threonine or tyrosine residue by a protein kinase can have a profound effect on the DNA binding ability, localization, protein/protein interaction and other activities of a transcription factor. In order to study the functional importance of serine 180 and serine 184 phosphorylation, we constructed a double mutant form of YY1 where both serines were changed to alanine, which is not phosphorylatable (Flag-YY1 S180,184A). In addition, we also constructed a mutant in which both serines were changed to aspartic acid, an attempt to mimic the negatively charged, phosphorylated state. HEK293 cells were transiently overexpressed with Flag-vector, Flag-YY1 wild type, Flag-YY1 S180,184A and Flag-YY1 S180,184D followed by nocodazole block. The cell lysates were analyzed by Western blot and probed with anti-pS184 antibody, then stripped and reprobed with anti-YY1 antibody. The double alanine mutant form of YY1 was not recognized by the p-S184 antibody (Fig. 5A), as seen previously (Fig. 1D); however the p-S184 antibody had partial affinity towards the double aspartic acid phospho-mutant of YY1 (Fig. 5A), indicating that the negatively charged aspartic acid was able to somewhat mimic the negative charge introduced by phosphorylation.

The first functional analysis we performed was a cell cycle progression assay. HEK293 cells were transiently overexpressed with Flag-vector, Flag-YY1 wild type, Flag-YY1 S180,184A and Flag-YY1 S180,184D for 48 hours, followed by propidium iodide staining and FACS analysis. No significant changes in the cell cycle were observed in cells overexpressing these mutant plasmids (Fig. S2A). We have previously reported that YY1 is mainly a nuclear protein during interphase of the cell cycle; as cells proceed into mitosis, YY1 becomes dispersed into the cytoplasm [7]. Monitoring the cellular localization of the phospho-mutant proteins of YY1 by immuno-staining did not show any differences between mutant and wild type YY1 and were mainly confined to the nucleus in interphase cells (Fig. S2B).

We then examined how the phosphomimetic mutant at both serine 180 and serine 184 of YY1 would affect its DNA binding activity. HEK293 cells were transfected with pCS2(+)Flag-vector, pCS2(+)Flag-YY1 wild type, pCS2(+)Flag-YY1 S180,184A and pCS2(+)Flag-YY1 S180,184D. Twenty four hours later, cell lysates were prepared and an electrophoretic mobility shift assay (EMSA) (Fig. 5B) and a Western Blot was performed (Fig. 5C). We used ^32^P-labeled H3.2α, P5-60 and p16 DNA double stranded oligonucleotides as probes ([56]–[58]). The H3.2α YY1 DNA binding site is located within the protein-encoding sequence of the histone 3.2 gene and is a YY1 transcriptional activating site [57]. The P5-60 YY1 DNA binding site is located in adeno-associated virus promoter and is also a YY1 transcriptional activating site [56]. The p16 YY1 DNA binding site is located in the p16 promoter and is a YY1 transcriptional repression site [58]. Interestingly, the YY1 S180,184D phospho-mutant showed an increase in DNA binding affinity with the YY1 activating sites (H3.2α and P5-60), whereas it had a decrease in binding affinity with the YY1 repressing site (p16) (Fig 5B).

### Phosphorylation/Acetylation Interplay in the Regulatory Domain of YY1

**Figure 6:**
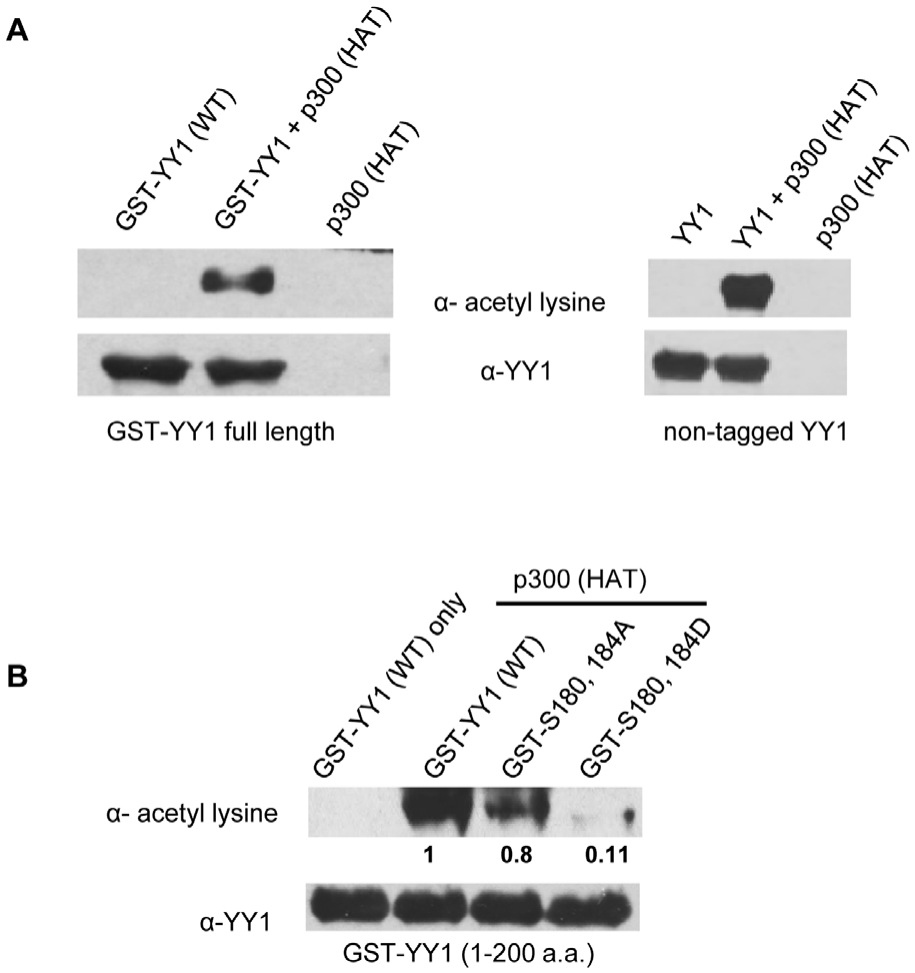
The histone acetyltransferase p300 acetylates full length YY1 wild type in vitro. (**A**) Cold in vitro acetylation assay reactions using purified p300-HAT domain and purified GST-YY1 (left panel) and non-tagged YY1 (right panel) as substrate. The reactions were performed at 30°C for 30 minutes followed by Western blot. The blot was probed with anti-acetyl-lysine antibody, then stripped and reprobed with anti-YY1 antibody. (**B**) Cold in vitro acetylation assay reactions using purified p300-HAT domain and purified GST-YY1 (1–200 a.a.) wild type, S180,184A and S180,184D deletion mutants. The reactions were performed at 30°C for 1 hour, followed by Western blot. The blot was probed with anti-acetyl-lysine antibody, then stripped and reprobed with anti-YY1 antibody.

It has been reported that the histone acetyltransferase p300 acetylates the lysine residues adjacent to both serine 180 and serine 184 (Fig. 1A). However, it was shown that only the truncated form of YY1 was able to be acetylated by p300, whereas, full length YY1 was not, possibly due to the conformation of YY1 protein [18]. Acetylation of YY1 has also been shown to play a critical role for YY1 transcriptional regulation [18]. In this study, we show that purified p300-HAT domain (Active Motif) can in fact efficiently acetylate both GST-tagged and non-tagged full length YY1 (Fig. 6A). More importantly, it also appears that phosphorylation of serine 180 and serine 184 interferes with p300 interaction and subsequent acetylation of the adjacent lysine residues in vitro, as seen in figure 6B. The phosphomimetic mutant of YY1 (GST-S180,184D) exhibited significantly less acetylation compared to both wild type YY1 and the double alanine mutant. Acetylation of the lysine residues adjacent to serine 184 by p300-HAT first, followed by phosphorylation by Aurora B did not show any effect in phosphorylation of serine 184 in vitro (data not shown).

## Discussion

The transcription factor YY1 has been shown to be a critical regulator in development and cell proliferation. A role in cell cycle control is one of the functions attributed to YY1 and it is involved in the transitions through the different phases of the cell cycle [2], [3], [57]. YY1 interacts with several key regulators of the cell cycle signaling pathways such as c-Myc, Retinoblastoma (Rb) protein and p53 [59]–[64]. Phosphorylation has been shown to occur on many transcription factors, including YY1. We have previously mapped multiple phosphorylation sites on YY1 (T39, S118, S247, T348, T378) [7], [23]–[25]. Polo like kinase 1 was identified as the first kinase for which YY1 was a substrate. It phosphorylates threonine 39 in the activation domain of YY1 at the G2/M stage of the cell cycle [25]. Casein kinase II α is another kinase which constitutively phosphorylates YY1 at serine 118. This modification prevents YY1 cleavage by caspase 7 during apoptosis [23].

In this study, we identified the kinase responsible for phosphorylating YY1 at serine 184, a novel phosphorylation site identified by a global mass spectrometry-based identification technique [44]. Based on the amino acid sequence of YY1 surrounding serine 184, the Aurora kinase family was one of the first candidates to show high probability for phosphorylation of YY1 at this site. To test this prediction, we performed a radioactive in vitro kinase assay with the different Aurora kinase isoforms. YY1 was phosphorylated by all three Aurora kinase (A, B and C) isoforms (Fig. 3A). A phospho-specific antibody that can recognize phosphorylated serine 184 on YY1 showed that only Aurora B was able to phosphorylate serine 184 in vitro (Fig. 3B and 3C), whereas, phosphorylation of YY1 by Aurora A and Aurora C occurs at other serine/threonine residues on YY1 that are yet to be identified. It was not surprising that Aurora B but not Aurora A phosphorylated serine 184. In a recent article by Kettenbach *et al.* [65], the difference between Aurora A and Aurora B kinase recognition motifs was analyzed. They showed that Aurora B exhibits a strong preference (55%) for basic amino acids immediately upstream and adjacent to the phosphorylation site ([R/K]p[S/T]). This was in contrast to potential Aurora A substrates that infrequently (9%) displayed a basic residue in this −1 position. Moreover, basic residues, and more frequently lysine rather than arginine, were found further upstream (positions −4 through −6) in the motifs present in the Aurora B cluster than were present in the motifs in the cluster for Aurora A [65]. It is striking that the amino acid residues surrounding serine 184, match almost perfectly to a consensus Aurora B phosphorylation motif (Fig. 1A).

We also identified two other kinases active in mitosis, PKA and ROCK1, that can phosphorylate YY1 at serine 184 in vitro (Fig. S1A and S1B). This might suggest the presence of multiple signaling pathways and multiple kinases that can phosphorylate YY1 at the same site. A similar pattern has also been observed with another Aurora B substrate, serine 10 in the tail of histone H3 (H3S10). This site in the histone tail has the sequence ([R][K]p[S]), which is comparable to the phosphorylation motif of serine 184 of YY1 ([K][K]p[S]). Phosphorylation of H3S10 can occur at different stages of the cell cycle by multiple kinases, depending on the context. In interphase, H3S10 phosphorylation correlates with chromatin relaxation and gene expression, whereas in mitosis it is associated with chromosome condensation [66]. Interestingly, all three kinases (Aurora B, PKA and ROCK1) which phosphorylate serine 184 of YY1 in vitro, also phosphorylate H3S10 [67]–[71]. However, inhibitors for PKA and ROCK1, did not inhibit phosphorylation at serine 184 in HEK293 cells. Therefore, in vivo phosphorylation of YY1 at serine 184 by a specific kinase, might be dependent on the context, and/or tissue and cell type.

The timing of the phosphorylation of serine 184 on YY1 occurs at the G2/M stage of the cell cycle (Fig. 2B), which correlates with the increase in expression and activation of Aurora B [28]. Aurora B activity is maintained throughout mitosis and cytokinesis. The majority of Aurora B substrates, including H3S10 are phosphorylated during this time in the cell cycle [34], [35], [39], [42], [43], [72]. Evidence that YY1 is a physiological substrate for Aurora B is strengthened by the physical interaction between YY1 and Aurora B shown by co-immunoprecipitation (Fig. 4C). Also, inhibition of phosphorylation by a specific Aurora inhibitor, VX-680 and Aurora B knockdown in HEK293 cells (Fig. 4A and 4B), clearly show that Aurora B is indeed the kinase responsible for serine 184 phosphorylation in vivo at G2/M.

An interesting finding in this study was the rapid dephosphorylation of serine 184 as cells exited mitosis and entered G1 stage of the cell cycle (Fig. 2C, right panel). In addition to the presence of highly active phosphatases at the end of mitosis [73], Aurora B is also known to be quickly degraded [74]. One of the major phosphatases that opposes and counteracts Aurora B kinase activity is protein phosphatase 1 (PP1) [53]–[55]. PP1 is also known to dephosphorylate H3S10 and many other Aurora B substrates [54]. In a cold in vitro kinase assay, we show that after YY1 phosphorylation at serine 184 by Aurora B, the addition of PP1, but not PP2A can efficiently dephosphorylate serine 184 (Fig. 3E). Dephosphorylation of YY1 at serine 184 by PP1 in vivo remains to be determined.

The functional significance of serine 180 phosphorylation, a possible in vivo phosphorylation site (Fig. 1D) and serine 184 phosphorylation, was also studied. Overexpression of a Flag-YY1 mutant, where both serine 180 and serine 184 were changed to alanine (non-phosphorylatable) or aspartic acid (phosphomimetic), did not show any significant differences in cell cycle progression of HEK293 cells, nor did we see any differences in cellular localization between the phosphomutants and wild type YY1. However, in an EMSA study, the phosphomimetic mutant exhibited an increase in DNA binding affinity with the YY1 activating sites (H3.2 and P5-60) and a decrease in binding with the YY1 repressing site (p16) (Fig. 5B). Even though serine 180 and 184 are not in YY1’s C-terminal zinc finger DNA binding domain, it appears that phosphorylation in the central regulatory domain can play an important role in specific DNA sequence recognition and binding. The timing of this phosphorylation at the G2/M stage of the cell cycle by Auorra B functionally overlaps with YY1 transcriptional regulation of genes at G2/M. More recently, the phosphorylation of the transcription factor p53 by Aurora B at two nearby sites (serine 269 and threonine 284) was shown to compromise p53 transcriptional activity [43]. Whether this phosphorylation of YY1 by Aurora B is important for transcriptional regulation remains to be determined.

YY1 has been shown to interact with numerous proteins, including transcriptional initiators (TFIIB, TBP and RNA polymerase II), transcriptional repressors (HDAC1, HDAC2 and HDAC4) and transcriptional activators and chromatin modifiers (CBP/p300 and PCAF) [18], [45], [75]–[79]. Most of these interactions with YY1 occur via the central glycine/alanine rich regulatory domain which includes both serine 180 and serine 184. It has been reported that the histone acetyltransferase p300 acetylates the lysine residues adjacent to both serines (Fig. 1A) and this modification can regulate YY1 transcriptional activity [18]. In this study, we show that purified p300-HAT domain acetylates both GST-tagged and non-tagged full length YY1 (Fig. 6A). More importantly, it also appears that phosphorylation of serine 180 and serine 184 inhibits the acetylation of YY1 by p300 (Fig. 6B). It is possible that the phosphorylation of these two residues in the regulatory domain of YY1 might affect protein/protein interaction with chromatin modifiers and therefore affect YY1 target gene expression. A unique interplay between acetylation and phosphorylation on adjacent residues has also been observed in other proteins, such as, estrogen receptor alpha (ERα), thymine DNA glycocylase (TDG) and histone H3[66], [80]–[82]. The interplay between these two different post-translational modifications was shown to have a significant impact on their respective function and activity. Whether a similar signaling cascade occurs in the process of posttranslational modification of YY1 and whether there are cellular mechanisms for coordination of YY1 phosphorylation and acetylation will be part of future studies.

**Figure 7:**
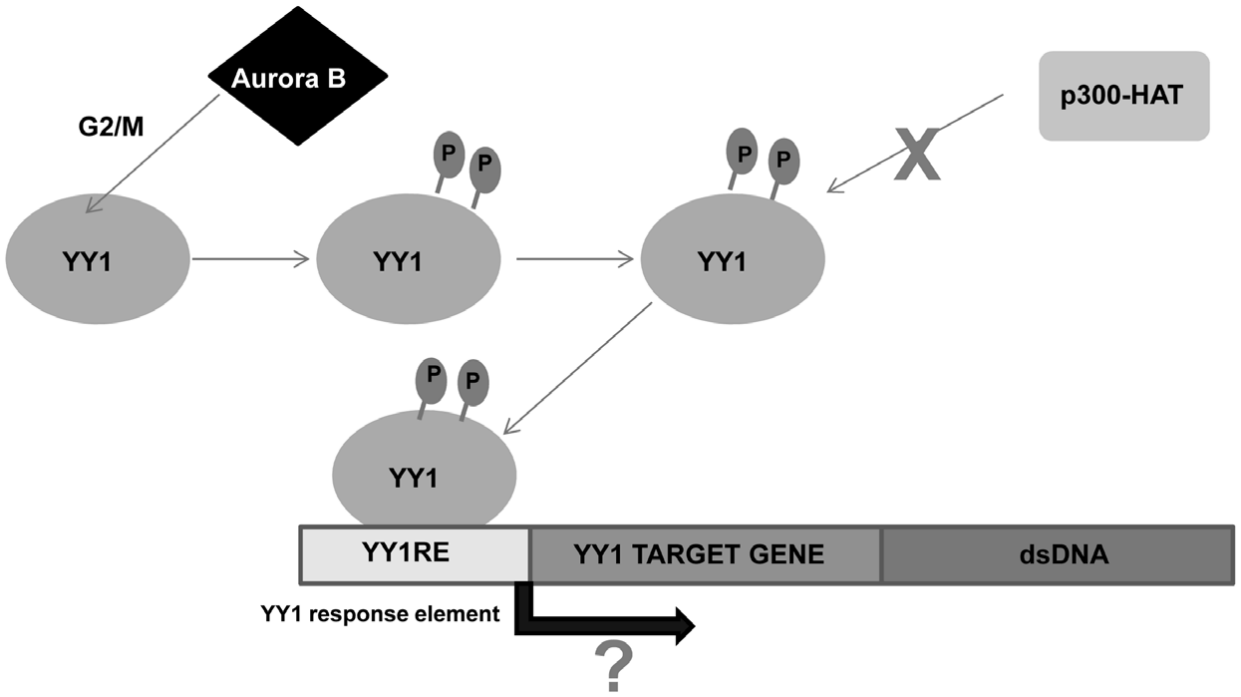
Schematic model of the regulation of YY1 by Aurora B at G2/M. YY1 is an Aurora B substrate at G2/M. Phosphorylation of YY1 in the regulatory domain prevents its association and acetylation by the histone acetyltransferase p300 and regulates YY1 DNA binding activity.

In summary, the findings of the present study identify YY1 as a novel substrate for the Aurora B kinase. Aurora B kinase phosphorylates YY1 on serine 184 and to a lesser extent serine 180 at the G2/M stage of the cell cycle (Fig. 7). We show that YY1 is rapidly dephosphorylated as the cells exit mitosis, likely by PP1. Also, our data indicates that phosphorylation at serine 180 and serine 184 can affect the DNA binding activity of YY1. However, the genes that are regulated by the phosphorylation of YY1 in this domain remain to be determined.

## Materials and Methods

### Cell Culture and Reagents

HEK293 and HeLa S3 cells (ATCC, Manassas, VA) were grown in DMEM (Cellgro, Herndon,VA) supplemented with 10% fetal bovine serum (FBS; Sigma, St. Louis, MO), 1% nonessential amino acids (Sigma, St. Louis, MO), and 1% Penicillin-Streptomycin (Mediatech). U2OS cells (ATCC, Manassas, VA) were cultured in McCoy’s 5A medium (Cellgro, Herndon,VA) supplemented with 10% FBS and 1% Penicillin-Streptomycin. All cells were grown at 37°C in 5% CO2. Cells were trypsinized and split into new plates at subconfluency. The Aurora kinase inhibitor, VX-680 (T-2304 Tozasertib, MK-0457), was purchased from LC Laboratories (Woburn, MA) and dissolved in DMSO at 10 mM stock concentration.

Whole Cell Extract (WCE) preparation, Immunoprecipitation (IP), Western blotting and Electrophoretic-Mobility Shift Assays (EMSA) were performed as previously described [7],[25],[57]. The antibodies used for IP were anti-HA (Covance, Princeton, NJ) and anti-Flag mouse mAb cross-linked to resin beads (Resin M2, Sigma); antibodies used for Western blotting were anti-YY1 (H-10, C-20, H414), anti-Cyclin B1, anti-acetyl-lysine (all Santa Cruz Biotechnology, Santa Cruz, CA) and anti-Aurora B (Abcam, Cambridge, MA). The rabbit polyclonal anti-pS184 was generated by New England Peptide using a synthesized phospho-peptide corresponding to amino acids 177–189 (Ac-GKKSGKK(p)SYLSGG-amide). Relative intensity of Western blot levels was quantitated using NIH ImageJ software (http://rsbweb.nih.gov/ij/).

### Plasmid Construction and Bacterial Expression of GST-YY1

GST-YY1 (1–200 a.a.) deletion mutant was constructed by digesting pGEX-2T-YY1 full length [25] with EcoRI/SmaI and religated after blunting the EcoRI site. GST-YY1 full length or GST-YY1 (1–200a.a.) deletion mutant, were overexpressed in bacterial cells as described previously [7].

### Cold in Vitro Kinase Assay

Non-tagged YY1, produced as previously described [25] or GST-YY1 attached to glutathione beads were used in cold in vitro kinase assays with purified Protein Kinase A (α and γ) catalytic domain, ROCK1, Plk1, PAK1 or Aurora (A,B and C) which were purchased from SignalChem (British Columbia, Canada). Kinase reactions were performed in kinase buffer (50 mM Tris pH 7.5, 150 mM NaCl, 10 mM MgCl_2_, 3 mM cold ATP) for 30 minutes at 30°C, with shaking. Reactions were then stopped by the addition of SDS-PAGE buffer and loaded for separation on a 10% SDS-PAGE gel.

For the kinase assays using HeLa and HEK293 whole cell extracts as the kinase source, 50 µg extracts were added to GST-YY1 attached to glutathione beads in kinase buffer supplemented with phosphatase inhibitors. After incubation, the GST-YY1 bead complexes were pelleted by centrifugation, and cell extracts were aspirated. Beads were washed 2X with kinase buffer, and then boiled in 4X SDS-PAGE loading buffer, prior to loading on the gel and subsequent Western blotting.

### Radioactive In Vitro Kinase Assay

Kinase reactions were performed in kinase buffer (50 mM Tris pH 7.5, 150 mM NaCl, 10 mM MgCl_2_, 50 µM ATP, 0.25 µM ^32^P-γ-ATP, 5 mM beta-glycerophosphate, 10 mM NaF, 1 mM DTT) for 30 minutes at 30°C, with shaking. GST-YY1 attached to glutathione beads were incubated with Aurora B, Protein Kinase A (γ) catalytic domain or ROCK1. Reactions were then stopped by the addition of 4X SDS-PAGE loading buffer and loaded for separation on a 10% SDS-PAGE gel. After staining with Coomassie Brilliant Blue R-250, to visualize the protein bands, gels were dried and exposed overnight to a Phosphorimager screen at room temperature. The screen was then scanned on a Typhoon 9410 imager (GE Healthcare, Waukesha, WI) for analysis.

### In Vitro Acetylation Assay

Acetylation reactions were performed in acetylation buffer (50 mM Tris pH 7.5, 50 mM KCl, 10 mM Na-Butyrate, 5% glycerol, 1 mM DTT and 1 mM Acetyl-CoA) for 1 hour at 30°C, with shaking. Non-tagged YY1 or GST-YY1 attached to glutathione beads were incubated with purified p300-HAT domain (Active Motif, Carlsbad, CA). Reactions were then stopped by the addition of SDS-PAGE buffer and loaded for separation on a 10% SDS-PAGE gel.

### In Vitro Phosphatase Assay

Immunoprecipitation (IP) of Flag-YY1 from HEK293 cells transiently overexpressing Flag-YY1 was performed using the anti-Flag mouse mAb cross-linked to resin beads (Resin M2, Sigma). WCEs were prepared and incubated with the antibody overnight, rotating at 4°C. Resin M2-Flag-YY1 complex was collected by centrifugation at 500 x *g* at 4°C for 2 min and then washed three times with lysis buffer and one additional time with lysis buffer without phosphatase inhibitors. Equal aliquots of the immuno-complex beads were then resuspended in phosphatase buffer (New England BioLabs, Beverly, MA) in the presence of 2 mM MnCl_2_, and incubated at 30°C, with or without λ-phosphatase (New England BioLabs, Beverly, MA) for 30 min. Reactions were then stopped by the addition of 4 X SDS-PAGE buffer, and loaded for separation on a 10% SDS-PAGE gel.

GST-YY1 beads phosphorylated by Aurora B were resuspended in phosphatase buffer in the presence of 2 mM MnCl2, and incubated at 30°C, with PP1 (New England BioLabs, Beverly, MA), PP2A (Millipore, Billerica, MA) or λ-phosphatase for 1 hour. Reactions were then stopped by the addition of 4 X SDS-PAGE buffer, and loaded for separation on a 10% SDS-PAGE gel.

### Plasmid Transfections

The pHM6-HA-Aurora A was a gift from Dr. Jin Cheng (Department of Molecular Oncology, Moffitt Cancer Center) [83]; pcDNA3-Flag-Aurora B was a gift from Dr. Mong-Hong Lee (Department of Molecular and Cellular Oncology, MD Anderson Cancer Center) [84]. HA-Aurora A, Flag-Aurora B and Flag-YY1 [7] were transiently overexpressed into HEK293 cells using Lipofectamine transfection reagent (Invitrogen, Carlsbad, CA) according to manufacturer’s instructions. Briefly, after equilibration with DMEM for 5 minutes, Lipofectamine was mixed with DNA in DMEM and incubated for 30 minutes prior to addition to HEK293 cells. After 6 hours, the medium/DNA/Lipofectamine mixture was replaced with fresh normal growth medium as described above.

### Aurora B siRNA

HEK293 cells were plated, cultured overnight, and then transfected with 20 nm control scrambled siRNA (Dharmacon, Chicago, IL) or Aurora B siRNA 5′-GGAAAGAAGGGATCCCTAAdTdT-3′ (Qiagen, Valencia, CA) [85]. The siRNA were transfected into HEK293 cells using DharmaFECT reagent (Dharmacon, Chicago, IL). After 48 hours of knockdown, the cells were lysed, and extracts were analyzed by Western blotting.

### Fluorescence-Activated Cell Sorter Analysis

HEK293 cells were trypsinized, washed two times with PBS, and then fixed in 70% ethanol on ice for at least 2 hours. After washing off the ethanol, cells were resuspended in propidium iodide (PI) solution (50 µg/ml PI, 200 µg/ml RNase A, 0.1% Triton-X 100 in PBS) and incubated for 30 min at 30°C. The cell suspension was then passed through a 50 µm nylon mesh to remove clumps. Cells were then analyzed based on DNA content on a fluorescence-activated cell sorter (FACS; FACS Canto; Becton Dickinson, San Jose, CA), and images were generated using BD FACS Diva software.

### Cell Synchronization

To synchronize HEK293 cells at G1/S, a double-thymidine arrest was performed as previously described [86]. For the double thymidine arrest/release experiment, cells were synchronized with 2.5 mM thymidine (Sigma) as described above, the cells were washed three times with PBS, one time with growth medium, and then released into fresh media. Samples were collected at the indicated time points. To synchronize cells at prometaphase, nocodazole (Sigma) was added to the medium at a final concentration of 100 ng/ml for 18 hours. For the nocodazole arrest/release experiment, cells were synchronized with nocodazole as described, then mitotic cells were detached from the plate surface by tapping the plate and collected by aspiration. Cells were washed two times with PBS and then one time with medium and replated in fresh growth medium. Samples were collected at indicated times for preparation of whole-cell extracts (WCEs).

### Mutagenesis

Point mutants of YY1 at serine 180 and serine 184 residues to aspartic acid and alanine were generated using QuikChange Lightning Site-Directed Mutagenesis Kit from Agilent Technologies (La Jolla, CA). Mutagenesis was performed according to manufacturer’s instructions, using the human YY1 open reading frame in pET-20 b(+)-YY1 plasmid [7] as a template. Primers were designed using the QuikChange Primer Design Program on the Agilent Technologies web site. All mutations were confirmed by sequencing. The mutated YY1 sequences were then subcloned into the pCS2(+) and pGEX-2T vectors as described previously [7], [25].

### Indirect Immunofluorescence

For indirect immunofluorescence, cells grown on coverslips were washed three times with PBS, fixed with 3.7% formaldehyde for 10 min RT, and then washed three times with PBS. Cells were permeabilized for 10 min at room temperature with PBS containing 0.2% Triton-X-100 and subsequently were washed three times with PBS. Immunostaining was performed by overlaying the coverslips with blocking solution (PBST, 1% IgG-free BSA) for 30 min at 37°C. Primary antibody was then added to the coverslips, in blocking solution, and incubated for 1 h at 37°C. Anti-Flag antibody (Sigma) was added at a final concentration of 1 µg/ml. Coverslips were then washed three times with PBST, and anti-rabbit Alexa-Fluor 546 (Molecular Probes, Eugene, OR) was then applied to the coverslips and incubated for 1 h at 37°C. After washing three times with PBST, cells were overlaid with DAPI solution (2 µg/ml in PBS) for 5 min, washed briefly, and mounted in Vectashield (Vector Laboratories, Burlingame, CA). Images were captured using a confocal microscope (Leica Microsystems, Exton, PA), taking 1 µm sections of the cells. The overlay images were generated using Leica LCS Lite Software.

## Supporting Information

Figure S1 **PKA and ROCK1 phosphorylate YY1 at serine 184 in vitro.** (A) Cold in vitro kinase assay reactions using purified PKA alpha, PKA gamma and ROCK1 kinases and purified non-tagged YY1 as substrate. The reactions were performed at 30°C for 30 minutes followed by Western blot. The blot was probed with anti-pS184 antibody, then stripped and reprobed with anti-YY1 antibody. (B) Radioactive in vitro kinase assay using purified PKA gamma and ROCK1 with GST-YY1 as substrate. The kinase reactions include GST-YY1 only (no kinase), kinase only (no substrate) and GSTY-YY1, GST-YY1 S180A, GST-YY1 S184A or GST-YY1 S180,184A with kinase. The reactions were performed as described in figure 3.
(PPTX)

Figure S2 **Cell cycle analysis and cellular localization of YY1 phospho-mutants in HEK293 cells.** **(A)** HEK293 cells were transiently transfected with Flag-vector, Flag-YY1 wild type, Flag-YY1 S180,184A and Flag-YY1 S180,184D for 48 hours. Cell cycle analysis of HEK293 cells after transfection was analyzed by fluorescence-activated cell sorting. Cells were stained with propidium iodide to analyze DNA content. Bar graphs show the percentage of HEK 293 cells in G1, S and G2/M with respect to total cell number. **(B)** HEK293 cells were transiently transfected with Flag-vector, Flag-YY1 wild type, Flag-YY1 S180,184A and Flag-YY1 S180,184D for 24 hours. Following transfection, cells were fixed and stained with anti-Flag antibody (red) followed by DAPI staining of DNA (blue).
(PPTX)
