# Supplementary material for: The Transcription Factor YY1 Is a Novel Substrate for Aurora B Kinase at G2/M Transition of the Cell Cycle
Source: PLoS One. 2012 Nov 30;7(11):e50645. doi: 10.1371/journal.pone.0050645 (PMC3511337; doi:10.1371/journal.pone.0050645)
Supplement: Figure S1 — PKA and ROCK1 phosphorylate YY1 at serine 184 in vitro. (A) Cold in vitro kinase assay reactions using purified PKA alpha, PKA gamma and ROCK1 kinases and purified non-tagged YY1 as substrate. The reactions were performed at 30°C for 30 minutes followed by Western blot. The blot was probed with anti-pS184 antibody, then stripped and reprobed with anti-YY1 antibody. (B) Radioactive in vitro kinase assay using purified PKA gamma and ROCK1 with GST-YY1 as substrate. The kinase reactions include GST-YY1 only (no kinase), kinase only (no substrate) and GSTY-YY1, GST-YY1 S180A, GST-YY1 S184A or GST-YY1 S180,184A with kinase. The reactions were performed as described in figure 3. (PPTX) [file pone.0050645.s001.pptx]

## Slide 1
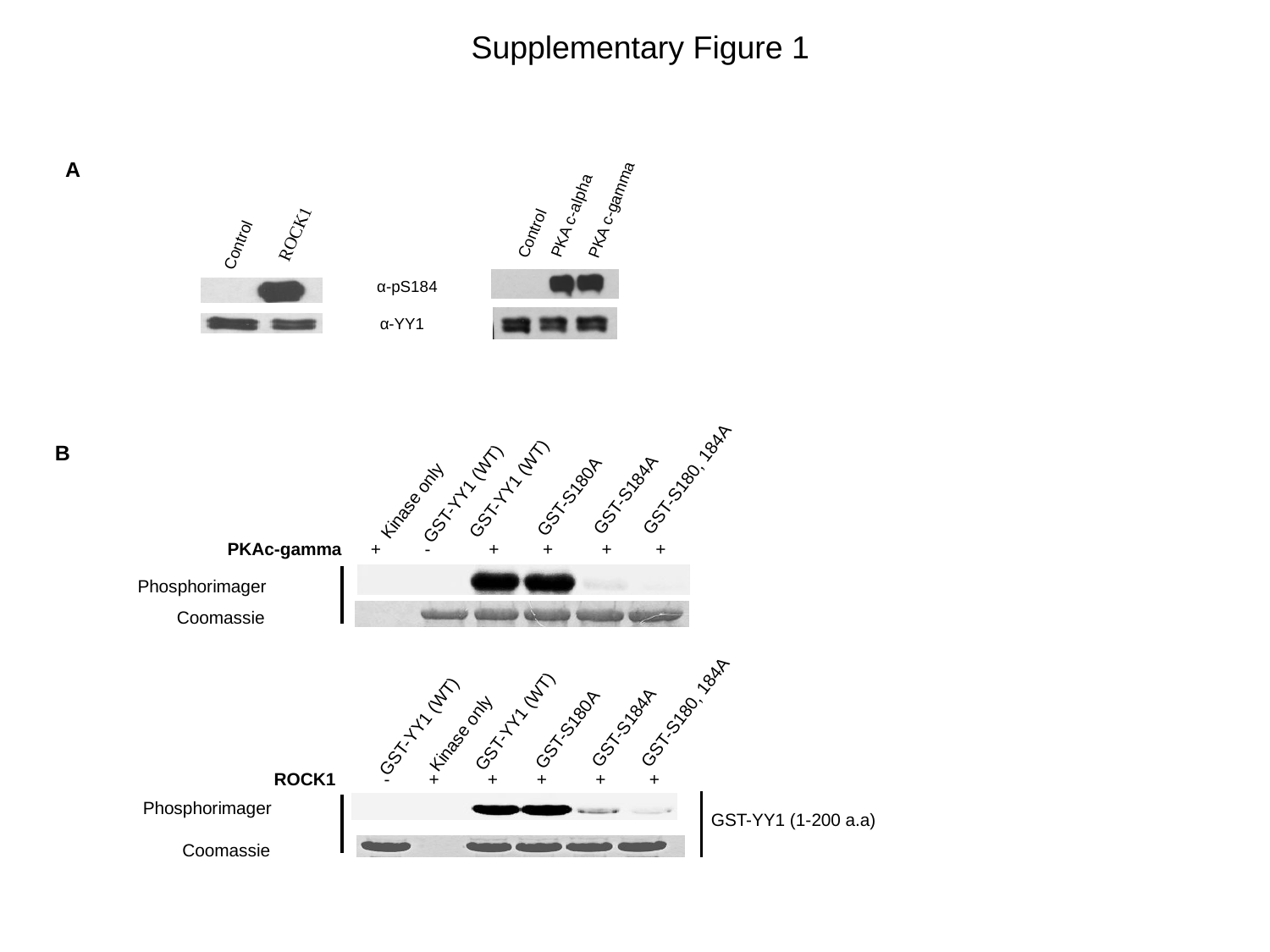

Supplementary Figure 1
A
PKA c-alpha
PKA c-gamma
ROCK1
Control
Control
α-pS184
α-YY1
B
GST-S180, 184A
GST-S184A
GST-S180A
GST-YY1 (WT)
Kinase only
GST-YY1 (WT)
PKAc-gamma + - + + + +
Phosphorimager
Coomassie
GST-S180, 184A
GST-S184A
GST-S180A
GST-YY1 (WT)
Kinase only
GST-YY1 (WT)
ROCK1 - + + + + +
Phosphorimager
GST-YY1 (1-200 a.a)
Coomassie
