# Supplementary material for: The Transcription Factor YY1 Is a Novel Substrate for Aurora B Kinase at G2/M Transition of the Cell Cycle
Source: PLoS One. 2012 Nov 30;7(11):e50645. doi: 10.1371/journal.pone.0050645 (PMC3511337; doi:10.1371/journal.pone.0050645)
Supplement: Figure S2 — Cell cycle analysis and cellular localization of YY1 phospho-mutants in HEK293 cells. (A) HEK293 cells were transiently transfected with Flag-vector, Flag-YY1 wild type, Flag-YY1 S180,184A and Flag-YY1 S180,184D for 48 hours. Cell cycle analysis of HEK293 cells after transfection was analyzed by fluorescence-activated cell sorting. Cells were stained with propidium iodide to analyze DNA content. Bar graphs show the percentage of HEK 293 cells in G1, S and G2/M with respect to total cell number. (B) HEK293 cells were transiently transfected with Flag-vector, Flag-YY1 wild type, Flag-YY1 S180,184A and Flag-YY1 S180,184D for 24 hours. Following transfection, cells were fixed and stained with anti-Flag antibody (red) followed by DAPI staining of DNA (blue). (PPTX) [file pone.0050645.s002.pptx]

## Slide 1
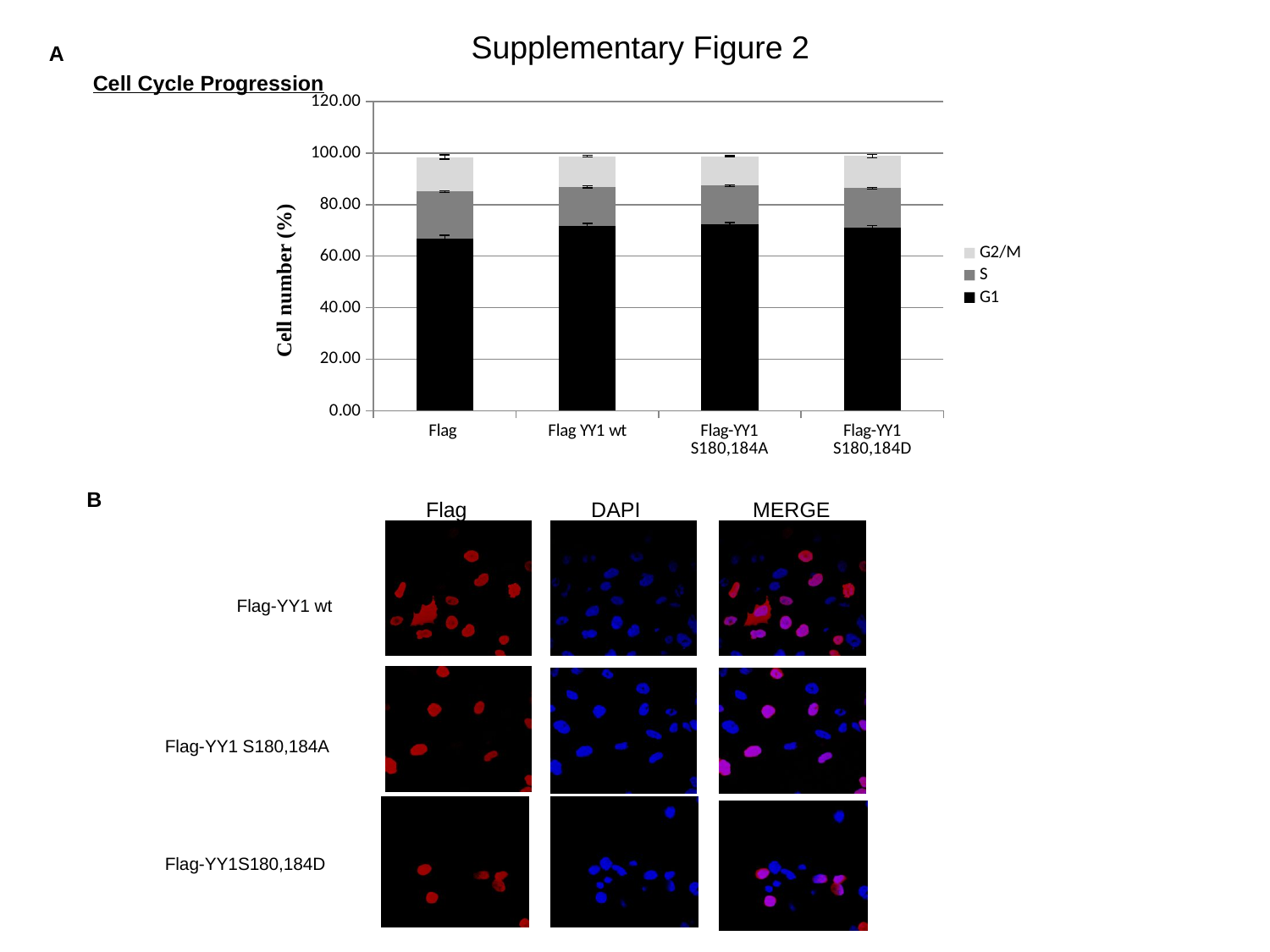

Supplementary Figure 2
A
Cell Cycle Progression
### Chart
| Category | G1 | S | G2/M |
|---|---|---|---|
| Flag | 66.76666666666667 | 18.266666666666666 | 13.399999999999999 |
| Flag YY1 wt | 71.76666666666667 | 15.166666666666666 | 11.833333333333334 |
| Flag-YY1 S180,184A | 72.46666666666665 | 14.9 | 11.333333333333334 |
| Flag-YY1 S180,184D | 71.16666666666667 | 15.200000000000001 | 12.466666666666667 |Cell number (%)
B
 Flag DAPI MERGE
Flag-YY1 wt
Flag-YY1 S180,184A
Flag-YY1S180,184D
